# *Materials* Best Paper Award 2013

**DOI:** 10.3390/ma6020609

**Published:** 2013-02-21

**Authors:** Maryam Tabrizian, Ophelia Han

**Affiliations:** 1 Department of Biomedical Engineering, Faculty of Medicine/Faculty of Dentistry, Duff Medical Science Building, McGill University, Room 313, 3775 University Street, Montreal, QC, H3A 2B4, Canada; E-Mail: maryam.tabrizian@mcgill.ca; 2 MDPI AG, Postfach, CH-4005 Basel, Switzerland and MDPI Branch Office, Beijing, China; E-Mail: ophelia.han@mdpi.com

*Materials* is instituting an annual award to recognize the outstanding papers in the area of materials science and engineering published in *Materials*.

We are pleased to announce the first “*Materials* Best Paper Award” for 2013. Nominations were selected by the Section Editor-in-Chiefs and Editorial Board members of *Materials* from all papers published in 2009. The awards are issued to reviews and articles respectively. We are pleased to announce that the following three papers were chosen:

**Article Award:**
  1^st^ Prize
    **Camilla Fredriksson, My Hedhammar, Ricardo Feinstein, Kerstin Nordling, Gunnar Kratz, Jan Johansson, Fredrik Huss and Anna Rising**
    Tissue Response to Subcutaneously Implanted Recombinant Spider Silk: An *in Vivo* Study
    *Materials* **2009**, *2*(4), 1908-1922; doi:10.3390/ma2041908
    Available online: http://www.mdpi.com/1996-1944/2/4/1908
  2^nd^ Prize
    **Nitar Nwe, Tetsuya Furuike and Hiroshi Tamura**
    The Mechanical and Biological Properties of Chitosan Scaffolds for Tissue Regeneration Templates Are Significantly Enhanced by Chitosan from *Gongronella butleri*
    *Materials* **2009**, *2*(2), 374-398; doi:10.3390/ma2020374
    Available online: http://www.mdpi.com/1996-1944/2/2/374
**Review Award:**
  **Sergey V. Dorozhkin**
    Calcium Orthophosphates in Nature, Biology and Medicine
    *Materials* **2009**, *2*(2), 399-498; doi:10.3390/ma2020399
    Available online: http://www.mdpi.com/1996-1944/2/2/399

The Editor-in-Chief, Prof. Dr. Maryam Tabrizian merits the article “Tissue Response to Subcutaneously Implanted Recombinant Spider Silk: An *in Vivo* Study” as an exceptional contribution to the field of natural biomaterials for scaffolding in tissue engineering. She ranks the paper on “The Mechanical and Biological Properties of Chitosan Scaffolds for Tissue Regeneration Templates Are Significantly Enhanced by Chitosan from *Gongronella butleri*” among the best published so far on the effect of chitsan structural parameters on the biological performance of this naturally occurring polymer.

Our review paper winner discussing “Calcium Orthophosphates in Nature, Biology and Medicine” is a part of a series of comprehensive reviews on calcium orthophosphate published in *Materials* which will greatly contribute to understanding the role played by calcium orthophosphates in a wide range of disciplines ranging from geology, chemistry, biology to medicine.

We believe that these three exceptional papers are valuable contributions to *Materials* and the scientific research area. On behalf of the Prize Awarding Committee and the Editorial Board of *Materials*, we would like to congratulate these three teams for their excellent work. In recognition of their accomplishment, for the article group, Dr. Anna Rising and Dr. Hiroshi Tamura will receive prizes of 600 CHF and 400 CHF, respectively, and the privilege of publishing an additional paper free of charge in open access format in *Materials*, after the usual peer-review procedure. While for the review group, Dr. Sergey V. Dorozhkin will receive the privilege of publishing a research article paper free of charge in *Materials* after the peer-review process.

Prize Awarding Committee
*Editor-in-Chief*, Section ‘Biomaterials’
  **Prof. Dr. Aldo R. Boccaccini**
  Institute of Biomaterials, Department of Materials Science and Engineering, University of Erlangen-Nuremberg, 91058 Erlangen, Germany
  E-Mail: aldo.boccaccini@ww.uni-erlangen.de
*Editor-in-Chief*, Section ‘Advanced Composites’
  **Prof. Dr. Luciano Feo**
  Department of Civil Engineering and Nano_Mates Center, University of Salerno, 84084 – Fisciano (SA), Italy
  E-Mail: l.feo@unisa.it
*Editorial Board Member*
  **Prof. Dr. Heather Sheardown**
  Department of Chemical Engineering, McMaster University, Room JHE-124A, 1280 Main Street West, Hamilton, Ontario, L8S 4L7, Canada
  E-Mail: sheardow@mcmaster.ca
*Editorial Board Member*
  **Prof. Dr. Rafael Luque**
  Departamento de Quimica Organica, Universidad de Cordoba, Campus de Rabanales, Edificio C-3 (Marie Curie-Anexo), Carretera Nacional IV-A, Km. 396, 14014–Cordoba, Spain
  E-Mail: q62alsor@uco.es
*Editorial Board Member*
  **Prof. Dr. Sergey Kustov**
  Departament de Física, Universitat de les Illes Balears, E-07122, Palma de Mallorca, Spain
  E-Mail: sergey.kustov@uib.es
*Editorial Board Member*
  **Prof. Dr. Walter Remo Caseri**
  Department of Materials, HCI H 509, Wolfgang-Pauli-Str. 10, CH-8093 Zürich, Switzerland
  E-Mail: walter.caseri@mat.ethz.ch

